# The *GLUT9* Gene Is Associated with Serum Uric Acid Levels in Sardinia and Chianti Cohorts

**DOI:** 10.1371/journal.pgen.0030194

**Published:** 2007-11-09

**Authors:** Siguang Li, Serena Sanna, Andrea Maschio, Fabio Busonero, Gianluca Usala, Antonella Mulas, Sandra Lai, Mariano Dei, Marco Orrù, Giuseppe Albai, Stefania Bandinelli, David Schlessinger, Edward Lakatta, Angelo Scuteri, Samer S Najjar, Jack Guralnik, Silvia Naitza, Laura Crisponi, Antonio Cao, Gonçalo Abecasis, Luigi Ferrucci, Manuela Uda, Wei-Min Chen, Ramaiah Nagaraja

**Affiliations:** 1 Laboratory of Genetics, National Institute on Aging, Baltimore, Maryland, United States of America; 2 Istituto di Neurogenetica e Neurofarmacologia, Consiglio Nazionale delle Ricerche, Cagliari, Italy; 3 Geriatric Rehabilitation Unit, Azienda Sanitaria Firenze, Florence, Italy; 4 Laboratory of Cardiovascular Science, National Institute on Aging, Baltimore, Maryland, United States of America; 5 Unità Operativa Geriatria, Istituto Nazionale Ricovero e Cura Anziani, Rome, Italy; 6 Laboratory of Epidemiology, Demography and Biometry, National Institute on Aging, Bethesda, Maryland, United States of America; 7 Center for Statistical Genetics, Department of Biostatistics, University of Michigan, Ann Arbor, Michigan, United States of America; 8 Clinical Research Branch, National Institute on Aging, Baltimore, Maryland, United States of America; University of Alabama at Birmingham, United States of America

## Abstract

High serum uric acid levels elevate pro-inflammatory–state gout crystal arthropathy and place individuals at high risk for cardiovascular morbidity and mortality. Genome-wide scans in the genetically isolated Sardinian population identified variants associated with serum uric acid levels as a quantitative trait. They mapped within *GLUT9*, a Chromosome 4 glucose transporter gene predominantly expressed in liver and kidney. SNP rs6855911 showed the strongest association (*p* = 1.84 × 10^−16^), along with eight others (*p* = 7.75 × 10^−16^ to 6.05 × 10^−11^). Individuals homozygous for the rare allele of rs6855911 (minor allele frequency = 0.26) had 0.6 mg/dl less uric acid than those homozygous for the common allele; the results were replicated in an unrelated cohort from Tuscany. Our results suggest that polymorphisms in *GLUT9* could affect glucose metabolism and uric acid synthesis and/or renal reabsorption, influencing serum uric acid levels over a wide range of values.

## Author Summary

High serum uric acid levels lead to gout and increase the risk of cardiovascular and kidney disease. To determine what genetic factors might contribute to uric acid levels, we conducted genome-wide scans of single nucleotide variations in DNA in population samples from Sardinia and Chianti. We report here that variants in the *GLUT9* gene are associated with altered uric acid levels in both populations. Unexpectedly, rather than being directly involved in uric acid synthesis or secretion, the *GLUT9* gene encodes a glucose transporter. It is of interest that the gene is predominantly expressed in liver, a major site of uric acid synthesis, and in kidney, where uric acid is excreted and reabsorbed. However, it now remains to be determined how altered glucose uptake can indirectly affect synthesis or excretion/reabsorption of uric acid, and whether *GLUT9* may provide a target for the therapeutic modification of uric acid levels.

## Introduction

Serum uric acid (UA) levels are frequently elevated in conjunction with several disorders, including obesity, hyperlipidemia, atherosclerosis, and hyperinsulinemia [1]. Most widely discussed have been findings of high serum UA in individuals at risk of cardiovascular disease [2,3]. High UA levels are causal in gout crystal arthropathy, and according to some authors, are associated with a negative cardiovascular risk profile. Elevated UA is a risk factor for all-cause and cardiovascular mortality in patients with prevalent cardiovascular disease, and an important prognostic factor in hypertension.

Identifying factors that affect UA levels could help our understanding of their role in pathology, improve clinical decisions about whether to treat moderate hyperuricemia [4,5], and help identify new targets for intervention aimed at managing undesirably high UA levels. UA levels in humans are generally higher than in other species because the gene for the primary enzyme that transforms UA in other species, uricase, is inactivated. Consequently, UA levels are determined by the balance between the production of UA through purine catabolism (the two important contributors being dietary purine levels and purines released from the DNA and RNA of damaged cells) and the rate of excretion/reabsorption in the kidney and intestine.

Several metabolic defects that may increase serum UA levels have been described. Changes in the level of UA synthesis can result from mutations or variation in activity of enzymes involved in purine metabolism. In one relevant instance, a microRNA has been reported that seems to modulate the synthesis of UA through its repression of phosphoribosyl pyrophosphate synthetase 1 [6]. Tissue ischemia can also stimulate UA production by the upregulation of xanthine oxidase. The alternative route to increased UA levels is a decline in UA clearance in the kidney. The urate/anion transporter [7] is highly expressed in the proximal kidney tubule, and genetic variants in the genes that code for the transporter may contribute to hyperuricemia [8].

In an attempt to discriminate genetic factors affecting UA levels, we scanned the genome with a panel of 362,129 single nucleotide polymorphisms (SNPs) in 4,305 Sardinian individuals. Here, we report association between SNPs found in a glucose transporter gene, *GLUT9*, and UA levels. We also show the association is confirmed in an independent population.

## Results

**Figure 1:**
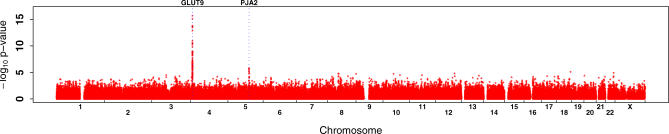
Genome-Wide Association Scan Results for UA Levels in Sardinian Cohort Probability of association (plotted as −log_10_) according to their positions along the p to q arms of each chromosome (1 to 22 and X) for each of 362,169 SNPs that passed quality control filters. The highest *p*-values are at the positions of the *GLUT9* and *PJA2* genes, and are indicated by vertical dotted lines.

**Figure 2:**
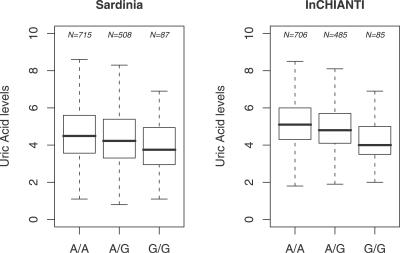
Boxplot for UA Levels Correlated with rs6855911 Genotypes in Sardinian and InCHIANTI Population Cohorts Standard boxplots are drawn with min, 0.25 quantile; median, 0.75 quantile; and 0.75 quantile + 1.5*IQR for the levels of UA (mg/dl). To increase readability, outliers were removed from the boxplots, but they were taken into account when estimating the parameters. Sample size for each genotype class is also annotated.

**Figure 3:**
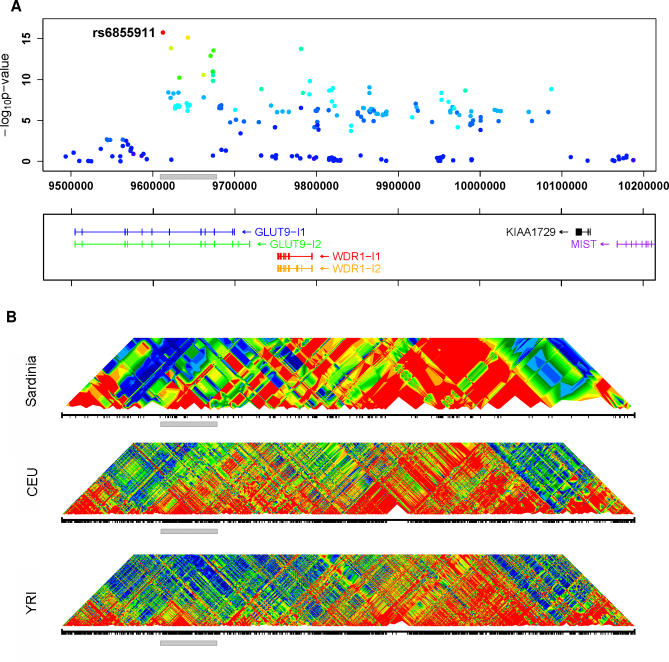
Association Results and Linkage Disequilibrium Patterns in Region Surrounding the *GLUT9* Gene (A) Summary of the association between SNPs in the region and UA levels. The SNP showing strongest association (rs6855911) is highlighted. Other SNPs are colored according to their degree of disequilibrium with rs6855911, ranging from high (red), to intermediate (green), to low (blue). Transcripts in the region are indicated at the bottom of the graph, with an arrow indicating the direction of transcription. (B) Patterns of linkage disequilibrium in the region in Sardinia and in two of the HapMap populations (CEU and YRI) [25], where *r* ^2^ values are calculated according to [26] and colored as (A). The grey bar marks the region of association and facilitates comparisons between the panels.

UA levels and genotypes were assessed for a cohort of individuals from Sardinia (see Materials and Methods). Using a Bonferroni threshold of 1.3 × 10^−7^ (0.05/362,129), we selected 38 SNPs potentially associated with UA levels. Unexpectedly, rather than identifying variants in purine catabolism or in the urate transporter, the scan pointed to *GLUT9* [9] on Chromosome 4 (Figure 1; Table S1). All SNPs exceeding the Bonferroni significance threshold map to the region that encodes this Class II glucose transporter [9]. The rare allele “G” of the top ranked SNP rs6855911 has an effect size of −0.317 mg/dl, with a more marked effect in women than in men (Table 1). Figure 2 shows the contributions of the two alleles of the SNP in homozygotes and heterozygotes. Their effect is observed across the full range of UA values. The strongest observed association of SNP rs6855911 and distribution of the SNPs in linkage disequilibrium are shown in Figure 3, along with the comparative linkage disequilibrium patterns in the HapMap Utah residents with ancestry from northern and western Europe (CEU) and Yoruba in Ibadan, Nigeria (YRI) samples.

**Table 1:**
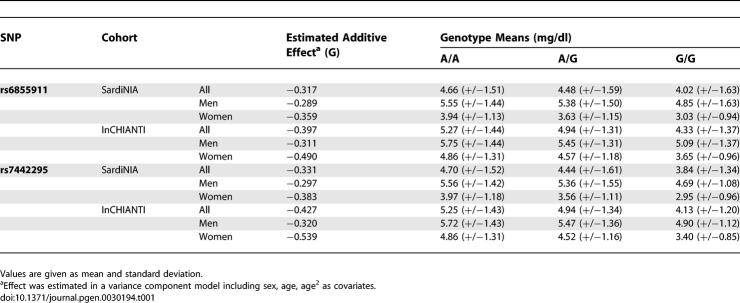
Effect Sizes of Two SNPs in *GLUT9* on UA Levels in Sardinian and InCHIANTI Cohorts

In a sequential analysis in which we selected the best SNP for each trait and then conditioned on it to successively select the next best SNP, a marker in the adjacent gene, *WDR1*, was selected at the second round, meaning that its signal is—at least in part—statistically independent from rs6855911 (Table 2). These results can support either the presence of multiple variants in the same region or reflect different degrees of linkage disequilibrium with a causal SNP that remains to be identified.

**Table 2:**
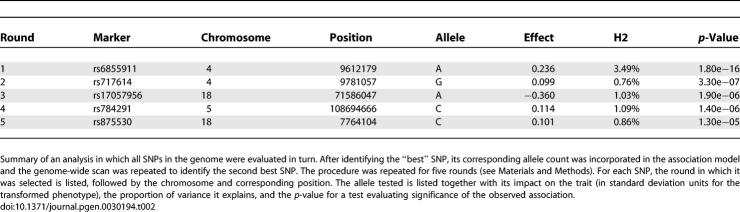
Association Signals Identified in a Sequential Analysis

The only other gene showing a cluster of SNPs associated with UA levels—though at far less striking *p*-values (on the order of 10^−7^)—is *PJA2*, a ubiquitin ligase gene on Chromosome 5 [10]; its position marked in Figure 1. This possible association has not been examined further.

Based on the genetic results, we hypothesized that variants underlying the association with UA levels affect a critical step in UA production or elimination. We started to test this hypothesis by studying the relationship between UA and the two top strongly associated SNPs in *GLUT9* in an independent population sample. We examined participants in the InCHIANTI study, an epidemiological study ongoing in two Italian towns in Chianti, Tuscany. A detailed description of the study design and data collection methods of the InCHIANTI study has been published [11,12]. The study population consists of 1,301 individuals (579 males and 722 females), comprising a random sample of the population aged 65 years and older supplemented with 30 men and 30 women randomly selected in each decade between ages 20 and 70. Table 1 and Figure 2 show that the results in this cohort replicate the findings in the Sardinian group. For SNP rs6855911, the allele G has a negative additive effect of 0.39 mg/dl (*p* = 1.54 × 10^−11^). Similarly, the rare allele G for rs7442295 has a negative additive effect of 0.42 mg/dl (*p* = 2.57 × 10^−12^). Again, *GLUT9* variants significantly and comparably affect UA levels over the whole distribution of values, without a threshold (Figure 2). Interestingly, while none of the Sardinian participants had hyperuricemia (UA > 7.5 mg/dl in men and >6.2 mg/dl in women), in the InCHIANTI study, participants with at least one G allele had a significantly lower prevalence of hyperuricemia (AA: 11.1%; AG: 7.2%; GG: 3.5%; *p* = 0.009).

UA levels generally increase with age (e.g., [13]), and the InCHIANTI cohort is considerably older than the Sardinian sample, resulting in higher mean values for each genotype (Figure 2). However, the values for corresponding age groups in the two are very similar, as are the contribution of the *GLUT9* alleles and the greater effect in females (Figure 2; Table 1).

Because the Sardinian population, as a founder population, is relatively homogeneous (reviewed in [13]), it can facilitate the detection of associated SNPs but might emphasize genes that are important only on the island. In this respect, mainland Italian residents are both genetically much more heterogeneous and distinct from Sardinians (as distant from Sardinians as American Caucasians are [14]). Thus, the replication of allele effects in Chianti suggests that *GLUT9* is involved in a general mechanism of UA production/elimination that may be biologically important in other populations as well.

The initial SNPs tested and found associated with UA levels all fall in noncoding regions. To assess any candidate SNPs in the coding region and/or promoter of the gene, the exons and a 1.5 kb presumptive promoter region upstream of the transcription start site were amplified by PCR and sequenced in 30 individuals (ten for each homozygote and ten heterozygotes for rs6855911). The primers used to amplify the genes are listed in Table S2. The sequencing found no new SNPs. However, we identified several SNPs in Sardinian DNA samples that were previously described in other populations. They include six SNPs in the promoter region and nine in exons (results are in Table S3). Among the other SNPs in the region, rs16890979, a nonsynonymous SNP in exon 8 that produces a Val253Ile amino acid change, was in strong linkage disequilibrium with rs6855911 (*r* ^2^ = 0.74 in the HapMap CEU and *r* ^2^ = 1.0 among the 30 resequenced individuals). However, genotyping of that coding SNP in a further 541 Sardinians suggests that rs6855911 and other noncoding SNPs in the region are more strongly associated with UA levels (*p* < 0.0004 for rs737267 and other noncoding SNPs in strong linkage disequilibrium with rs6855911, *p* = 0.02 for rs16890979). Further analyses are necessary to identify the causal variant(s) in the region.

## Discussion

How might variants of *GLUT9* differentially affect UA levels? Studies have verified that GLUT9 transports glucose [15], and both characterized isoforms, comprising 540 and 511 amino acids [15], respectively, are highly expressed in liver and distal kidney tubules. In the liver, where much UA is synthesized, rare deficiency of glucose-6-phosphatase (Glycogenosis Type I; Online Mendelian Inheritance in Man 232200) leads to increased levels of UA. Variations in the uptake of glucose via *GLUT9* might also result in increases or decreases in glucose-6-phosphate and modulate metabolism through the pentose phosphate shunt. Augmented levels of phosphoribosyl pyrophosphate synthesis, for example, could then lead to increased hepatic production of UA.

Alternatively, or in addition, the effect of *GLUT9* on UA level may be exerted through effects on renal excretion. In the kidney, urate transport occurs in the proximal tubular epithelium, whereas *GLUT9* is expressed in more distal nephron segments, perhaps the distal convoluted or connecting tubules [16]. However, those segments are relatively anaerobic, and one can speculate that the levels of lactate and other anions could be altered by metabolism from the glucose supplied by *GLUT9*, with consequent changes in organic anion concentration in the interstitium and neighboring proximal tubule cells. Anions are exchanged for urate by the transporter *URAT1*, with lactate as a preferred exchange ion [7], affecting the coupled process whereby filtered urate is reabsorbed from the urine in exchange for cytosolic organic anions. Hence, *GLUT9* variants in the kidney could change the ambient level of organic anions and thereby alter urate reabsorption and circulating urate levels. Effects could be more marked in individuals with purine-rich diets, or in hypertensive individuals in whom reabsorption is favored by reduced blood flow in the proximal tubule [17] and/or localized ischemia [2].

Regardless of underlying mechanism, *GLUT9* variants clearly have a significant modulating effect on levels of UA. Homozygotes for the two allelic variants differ in serum UA by 0.6 mg/dl, or on the order of 10% of the mean levels. Based on the findings in an independent study [18], such a difference in UA levels would change the rate of cardiovascular events by about 1%.

In further assessing the impact of such variation, it is notable that many authors ([19] and review in [4]) have pointed out that UA can function as an antioxidant, and that at earlier times in evolution, this may have been beneficial to human populations, in fact leading to the inactivation of uricase. Consistent with a possible need for UA in past generations, we found that the observed allele associated with higher levels of UA, AA, is more common on a population level. Allele GG, which is associated with lower levels, occurs in only 6.9% of the Sardinian and 6.6 % of the InCHIANTI population sample (Table S4 gives the absolute numbers).

In those individuals with intermediate levels of UA, it remains unclear whether it functions simply as an antioxidant that tends to increase in conditions characterized by tissue damage or whether it has a deleterious effect; and at very high levels UA is patently pathological. However, in individuals with very high levels, UA is patently pathological and mortality increases [3]. In such individuals, the G allele may be partially protective, and intriguingly, in the InCHIANTI study, participants with at least one G allele had a significantly lower prevalence of hyperuricemia (defined as UA >7.5 mg/dl in men and >6.2 mg/dl in women) compared to those with only A alleles. It would be of interest to verify whether variants of associated SNPs in the *GLUT9* gene are less represented in particular groups of individuals with different clinical forms of hyperuricemia and in sporadic gout patients. Such effects would support the suggestion that *GLUT9* may eventually provide a target for clinical intervention.

## Materials and Methods

### Recruitment and UA determinations.

We recruited and phenotyped 6,148 individuals, male and female, 14–102 y old, from a cluster of four towns in the Lanusei Valley [13]. Blood samples were collected in the morning after the participants had been fasting for at least 12 h and after sitting for 15 min, both for SardiNIA and InCHIANTI cohorts. Aliquots of serum were immediately obtained and stored at −80 °C, and were subsequently used for the assessment of UA. UA (mg/dl) was measured using enzymatic–colorimetric methods (Bayer for SardiNIA; Roche Diagnostics for INCHIANTI). The lower limits of detection were 0.2 mg/dl, range 0.2–25.0 mg/dl, intra-assay and inter-assay coefficients of variation were equal to 0.5% and 1.7%, respectively. Hyperuricemia was defined as a serum urate concentration >7.5 mg/dl (450 μmol/l) in men and >6.2 mg/dL (372 μmol/l) in women, in agreement with clinical laboratory standards.

### Genotyping.

In the Sardinian cohort, 3,329 and 1,412 individuals were genotyped with the Affymetrix 10K and Affymetrix 500K Mapping array set, respectively, with 436 individuals generating an overlapping dataset. We took advantage of the relatedness among individuals in our sample to reduce study costs. Using a modified Lander-Green algorithm, full genotypes on the 2,893 individuals typed with only the 10K panel were imputed based on stretches of shared haplotype, permitting analyses on 4,305 individuals [20].

For genotyping for replication in the InCHIANTI cohort, allelic discrimination assays with predeveloped assay reagents (Applied Biosystems) for SNPs rs6855911 and rs7442295 were carried out using an ABI 7900HT sequencer detector system. Each 10 μl reaction volume was prepared according to manufacturer's directions, and contained 5 μl of twice-concentrated TaqMan Universal PCR master mix, 0.25 μl of 40-fold concentrated SNP genotyping assay mix with amplifying primers and oligonucleotide labeled with VIC or FAM reporter dyes for the corresponding alleles, 1 μl containing 5 ng of template DNA from an InCHIANTI subject, and 3.75 μl of water. Assays were run in 96-well format. Controls included DNA with sequence-verified homozygous alleles, DNA with heterozygous alleles, or no template DNA, in each assay plate. Following allele discrimination assays, a total of 14 random samples representing each allelic state were also checked by direct sequencing, confirming allele calls in all cases.

### Statistical analyses.

Analyses in the Sardinia cohort were carried out as in [20]. We focused on 362,129 SNPs that passed quality control checks. We used a linear regression model to estimate the association between each SNP and UA levels. To avoid inflated type I error rates due to the departure from normality of measured UA levels, we applied an inverse normal transformation to the phenotype prior to analysis. We used an additive model and included sex, age, and age^2^ as covariates.

For individuals who had genotype data available at the SNP being tested, we coded genotypes as 0, 1, or 2, depending on the number of copies of an arbitrary reference allele for each SNP. For individuals with missing genotype data, we used the Lander-Green algorithm to estimate the number of copies of the allele carried by each individual (based on the genotypes of family members) and assigned each individual a score ranging between 0 and 2 [21]. This estimate incorporates allele frequency information, the genotypes of relatives for the SNP of interest, and flanking marker data. For computational efficiency, the Lander-Green algorithm was applied to sub-pedigrees, each including no more than 20–25 individuals, resulting in a dataset where the average analysis unit consisted of a family with 12.3 members and 3.2 generations. Finally, we used a variance component approach to account for correlation between different observed phenotypes within each family [21]. To evaluate association on the X chromosome, we modeled a polygenic variance component shared according to an X-linked kinship coefficient in addition to the usual autosomal polygenic variance component [13,21]. Further, we assumed that average phenotypic values for hemizygous males would be the same as for homozygous females [13,21].

Analysis for replication in the InCHIANTI population was carried out by again coding genotypes as 0, 1, or 2 and fitting a linear regression model adjusting for sex, age, and age^2^.

For the sequential analysis, the best SNP for each trait was selected first, and the analysis was conditioned on it to select successively the next best SNP. To carry out this conditioning, we simply added the best SNP as a covariate to the model and repeated the genome-wide association scan for all other SNPs. The same process was repeated in successive rounds. This sequential analysis can help identify regions with multiple independent association signals. After five rounds, additional SNPs identified had *p*-values that were far less significant.

### Sequencing of exons and putative promoter regions with SNP variants.

Oligonucleotide primers to amplify putative promoter regions and exons, including the splice sites for two isoforms of the *GLUT9* gene, were designed based on the exon–intron borders represented in the University of California Santa Cruz (UCSC) genome assembly [22] using the primer prediction program Primer3 [23] interfaced with the UCSC genome browser. In the putative promoter regions, repeats were masked by Repeat Masker [24], and only primers outside the repeats were selected. A list of primers used to amplify the exons is given in Table S2.

The exons were amplified by PCR in 25 μl reactions containing 0.2 mM dNTPs, final concentration 1 μM each of forward and reverse primers, 2 mM MgCl_2_, and 50 ng of genomic DNA as template, with 1 unit recombinant Taq DNA polymerase (Invitrogen). The cycling conditions were as follows: 95 °C 5 min, followed by 95 °C for 30 sec, 60 °C for 30 sec, and 72 °C for 30 sec for 30 cycles. The PCR products were purified by using ExoSAP-IT (USB Corporation) and were sequenced bidirectionally using the ABI PRISM BigDye Terminator v3.1 Cycle Sequencing Kit according to the manufacturer's recommendations, on an ABI PRISM 3100 Sequencer, and visualized with DNA Genetic Analyzer software (ABI PRISM 3100 Genetic Analyzer).

## Supporting Information

Table S1 SNPs with Genome-Wide *p*-Value <10^−6^ for an Effect on UA Level in the Sardinian Cohort The 40 SNPs all fall in or very near the *GLUT9* gene; positions of the sequences are according to UCSC [22], March 2006 genome build.
(62 KB DOC)

Table S2 Primers Used to Amplify Each Exon and Putative Promoter Region of *GLUT9*
Forward and reverse primers are listed for exons and a 1.5 kb putative promoter regions of the *GLUT9* isoform 2. Positions along the gene are according to UCSC [22] March 2006 genome build. Dashes indicate exons with no polymorphic SNPs in the database.
(30 KB DOC)

Table S3 Sequence Variants in *GLUT9* Putative Promoter, Exons, and Introns in Sardinian Individuals with Alternative Genotypes for SNP rs6855911 DNA segments from ten individuals homozygous for the minor allele (GG), the major allele (AA), or heterozygous (AG) for rs6855911 were amplified by PCR and sequenced. For each individual, using primers listed in Table S1, sequence was determined for 1.5 kb putative promoter region for isoform 2, exons, and intron/exon boundaries of *GLUT9*. SNPs [25] with the indicated identification numbers were found in the samples, and genotypes are shown for each. An asterisk marks the strongest candidate for association. SNPs in putative promoter regions and exons are as in Table S1. SNP rs3733589 in exon 4 was monomorphic in the samples tested (unpublished data). Amino acid positions in the protein refer to protein accession number NP001001290. The Gly 25 Arg change is specific to *GLUT9* isoform I (NP064425). nd, allelic calls not determined
(67 KB DOC)

Table S4 Numbers of Individuals with Each Genotype for Two SNPs in Sardinia and InCHIANTI Population Cohorts (26 KB DOC)

### Accession Numbers

The National Center for Biotechnology Information (NCBI) Online Mendelian Inheritance in Man (OMIM, http://www.ncbi.nlm.nih.gov/entrez/dispomim.cgi?id=232200) accession number for Glycogen storage disease Ia, simulating primary gout and xanthomatosis, is MIM 232200.

The NCBI Entrez database (http://www.ncbi.nlm.nih.gov/sites/gquery) accession number for Solute carrier family 2, member 9 protein isoform I, is NP 064425.

## Abbreviations

*CEU*: Utah residents with ancestry from northern and western Europe
GLUT9: glucose transporter 9
UA: uric acid
YRI: Yoruba in Ibadan, Nigeria
