# Supplementary material for: The GLUT9 Gene Is Associated with Serum Uric Acid Levels in Sardinia and Chianti Cohorts
Source: PLoS Genet. 2007 Nov 9;3(11):e194. doi: 10.1371/journal.pgen.0030194 (PMC2065883; doi:10.1371/journal.pgen.0030194)
Supplement: Supplementary file 1 [file pgen.0030194.st001.doc]

**Supplemantary Table 1. SNPs with genome-wide p-value <10^-6^ for UA in the Sardinian cohort.**

| **SNP** | **Chr** | **position** | **allele** | **freq** | **effect** | **H2** | **p-value** |
| --- | --- | --- | --- | --- | --- | --- | --- |
|  |  |  |  |  |  |  |  |
| rs6855911 | 4 | 9612179 | A | 0.74 | 0.236 | 3.49% | 1.85E-16 |
| rs7442295 | 4 | 9642649 | A | 0.80 | 0.245 | 3.15% | 7.68E-16 |
| rs4697700 | 4 | 9622061 | G | 0.78 | 0.224 | 2.85% | 5.52E-15 |
| rs717614 | 4 | 9781057 | G | 0.63 | 0.154 | 1.79% | 6.01E-15 |
| rs7669607 | 4 | 9674070 | C | 0.82 | 0.241 | 2.77% | 2.87E-14 |
| rs6449213 | 4 | 9670484 | A | 0.85 | 0.249 | 2.60% | 1.29E-13 |
| rs13111638 | 4 | 9673159 | G | 0.84 | 0.220 | 2.08% | 4.85E-12 |
| rs4481233 | 4 | 9632348 | G | 0.86 | 0.226 | 2.06% | 1.19E-11 |
| rs7680126 | 4 | 9661865 | A | 0.79 | 0.204 | 2.26% | 2.75E-11 |
| rs3733588 | 4 | 9673572 | A | 0.74 | 0.188 | 2.19% | 2.97E-11 |
| rs4529048 | 4 | 9673381 | T | 0.74 | 0.179 | 1.99% | 7.27E-11 |
| rs12498927 | 4 | 9791792 | C | 0.60 | 0.159 | 1.99% | 1.61E-10 |
| rs17198547 | 4 | 9817688 | G | 0.79 | 0.180 | 1.72% | 7.29E-10 |
| rs7689060 | 4 | 9981732 | G | 0.80 | 0.182 | 1.71% | 8.03E-10 |
| rs4697714 | 4 | 9820055 | A | 0.79 | 0.182 | 1.77% | 1.17E-09 |
| rs7671266 | 4 | 9732645 | C | 0.80 | 0.188 | 1.82% | 1.48E-09 |
| rs4698050 | 4 | 10087017 | C | 0.79 | 0.181 | 1.77% | 1.50E-09 |
| rs956311 | 4 | 9864650 | T | 0.58 | -0.112 | 0.99% | 1.72E-09 |
| rs12509714 | 4 | 9783360 | G | 0.63 | 0.153 | 1.76% | 2.11E-09 |
| rs10939723 | 4 | 9815374 | G | 0.79 | 0.180 | 1.72% | 2.34E-09 |
| rs4697698 | 4 | 9618846 | C | 0.54 | -0.145 | 1.70% | 3.83E-09 |
| rs7375599 | 4 | 9631187 | C | 0.54 | -0.145 | 1.70% | 4.17E-09 |
| rs10489070 | 4 | 9952621 | C | 0.79 | 0.172 | 1.61% | 4.32E-09 |
| rs998675 | 4 | 9625098 | A | 0.55 | -0.145 | 1.69% | 5.13E-09 |
| rs4604059 | 4 | 9791334 | C | 0.61 | 0.145 | 1.62% | 6.52E-09 |
| rs956312 | 4 | 9864595 | T | 0.58 | -0.112 | 0.98% | 7.97E-09 |
| rs17246501 | 4 | 9661979 | A | 0.53 | -0.141 | 1.60% | 1.54E-08 |
| rs4235346 | 4 | 9621565 | T | 0.53 | -0.145 | 1.70% | 1.97E-08 |
| rs12510549 | 4 | 9952736 | A | 0.78 | 0.160 | 1.45% | 3.07E-08 |
| rs17251963 | 4 | 9818830 | A | 0.79 | 0.159 | 1.37% | 4.85E-08 |
| rs4698009 | 4 | 9961262 | A | 0.78 | 0.162 | 1.46% | 5.37E-08 |
| rs6839490 | 4 | 9641269 | G | 0.55 | 0.133 | 1.42% | 6.39E-08 |
| rs4640669 | 4 | 9822002 | G | 0.77 | 0.152 | 1.34% | 6.43E-08 |
| rs9291642 | 4 | 9683544 | T | 0.91 | 0.208 | 1.12% | 8.33E-08 |
| rs6449178 | 4 | 9644953 | G | 0.57 | 0.131 | 1.38% | 9.77E-08 |
| rs9291640 | 4 | 9683355 | T | 0.84 | 0.166 | 1.23% | 1.14E-07 |
| rs7658170 | 4 | 9642862 | T | 0.56 | 0.132 | 1.40% | 1.20E-07 |
| rs4455410 | 4 | 9629566 | T | 0.55 | 0.130 | 1.35% | 1.23E-07 |
| rs4311316 | 4 | 9632240 | C | 0.55 | 0.129 | 1.34% | 1.42E-07 |
| rs7378340 | 4 | 9631467 | C | 0.56 | 0.129 | 1.34% | 1.49E-07 |
