# Supplementary material for: The GLUT9 Gene Is Associated with Serum Uric Acid Levels in Sardinia and Chianti Cohorts
Source: PLoS Genet. 2007 Nov 9;3(11):e194. doi: 10.1371/journal.pgen.0030194 (PMC2065883; doi:10.1371/journal.pgen.0030194)
Supplement: Supplementary file 2 [file pgen.0030194.st002.doc]

Supplementary Table 2: List of primers used to amplify and sequence putative promoter regions and exons

| **Targeted Region** | **5’ Primer** | **3’ Primer** | **Product Size(bp)** | **SNP ID** |
| --- | --- | --- | --- | --- |
| Putative promoter isoform 1 | GAAGGGAAGACTGTTCTTGG | CTAGTATCAGAAGCCTGGAG | 705 | rs13137343 rs13101785 rs13137074 rs7349721 |
| Putative promoter isoform 2 | GGGATGGAAGATTTTTGAGC | ACTCAGCCAACAGGAACTGAGTG | 765 | rs7679916 rs10516197 |
| Exon 1a | TTCTTTGCTTGGCATTACCC | GCCTTGGCATTCAACATTC | 324 | -- |
| Exon 2 | GCAAACCTTTCGAAGACTGC | TTAACAGCCTCCCACTGACC | 292 | rs6449237 rs6820230 |
| Exon 1b | TCAAATTGCCACACTTCCTG | GCCTGCCTTCCCCACAG | 331 | rs2276961 |
| Exon 3 | GGACAAAGACTTCTCCTCCG | CGCACCCGAAGGTTTCC | 274 | -- |
| Exon 4 | TTGTTTTGTACACTGGCTTGC | GGACCCTGACAATGACACAG | 309 | rs13113918 rs10939650 |
| Exon 5 | CTGGGATGGACAGTTCAGTG | CTCACATTTTGGGACACCC | 259 | rs3733589 |
| Exon 6 | GTGCTATTTAATGTCCAGGGC | GCAAAATACAGGGACCAGCTT | 351 | rs13125646 |
| Exon 7 | CAGTCCCCTCAACAATGACC | ATTGGCCCAGGTCCCAG | 272 | -- |
| Exon 8 | CAGGGCCCAGCATTAGAC | CACCCTCTGATCCCTCCAG | 312 | rs16890979, rs3733591 |
| Exon 9 | AGAAAATAGATTAAGTCCTTCCACTG | TGAACATTCCCACTGTGCTG | 238 | rs2280205 |
| Exon 10 | CCCTGTTGTGGTTAGAAATGG | CGATGAAGCCAGAAGTCAGC | 223 | -- |
| Exon 11 | ACTCAGGTCCTCCGGTCAC | AGCTTGGTGATGCCATGAG | 193 | -- |
| Exon 12 | GTGTGGCAGATGGAGATGG | AGTGCTGCAGAATCAAAGGG | 262 | -- |
| Exon 13 | TTGGTATATGAATGTGGAGCTATC | TTCCAGAAGGGTTTGGGAG | 535 | -- |
