# Supplementary material for: The GLUT9 Gene Is Associated with Serum Uric Acid Levels in Sardinia and Chianti Cohorts
Source: PLoS Genet. 2007 Nov 9;3(11):e194. doi: 10.1371/journal.pgen.0030194 (PMC2065883; doi:10.1371/journal.pgen.0030194)
Supplement: Supplementary file 3 [file pgen.0030194.st003.doc]

**Supplementary Table 3 : Genotypes for SNPs found in exons and putative promoter sequence for the two *GLUT9* isoforms**

| Genotype of rs6855911 | | | GG | | | | | | | | | | AG | | | | | | | | | | AA | | | | | | | | | |
| --- | --- | --- | --- | --- | --- | --- | --- | --- | --- | --- | --- | --- | --- | --- | --- | --- | --- | --- | --- | --- | --- | --- | --- | --- | --- | --- | --- | --- | --- | --- | --- | --- |
| SNP | DNA sequence variant | Aminoacid change |  | | | | | | | | | |  | | | | | | | | | |  | | | | | | | | | |
| rs13137343 | G>T | - | TT | GG | GT | GT | GT | GT | GG | GG | GG | GG | GT | GT | GG | GT | GG | GT | GT | TT | GT | TT | TT | TT | GT | GT | TT | TT | GT | TT | TT | TT |
| rs13101785 | A>T | - | TT | AA | AT | AT | AT | AT | AA | AA | AA | AA | TT | AT | AA | AT | AA | AT | AT | TT | AT | TT | TT | TT | AT | AT | TT | TT | AT | TT | TT | TT |
| rs13137074 | G>A | - | AA | GG | GA | GA | GA | GA | GG | GG | GG | GG | AA | GA | GG | GA | GG | GA | GA | AA | GA | AA | AA | AA | GA | GA | AA | AA | GA | AA | AA | AA |
| rs7349721 | T>A | - | AA | AA | AA | AA | TA | TA | TA | TA | AA | AA | AA | TA | AA | AA | AA | TA | AA | AA | TA | AA | AA | AA | AA | AA | AA | AA | AA | AA | AA | AA |
| rs7679916 | G>A | - | AA | GG | GA | GA | GA | GA | GG | GG | GG | GG | AA | GA | GG | GA | GG | GA | GA | AA | GA | AA | AA | AA | GA | GA | AA | AA | GA | AA | AA | AA |
| rs10516197 | G>A | - | GG | GG | GG | GG | GA | GG | GA | GG | GG | GG | GG | GG | GG | GG | GG | GG | GG | GG | GG | GG | GG | GG | GG | GG | GG | GG | GG | GG | GG | GG |
| rs6449237 | T>C | - | TT | CC | TC | TC | TT | TT | TT | TC | CC | CC | TT | TC | CC | TC | CC | TT | TC | TT | TT | TT | TT | TT | TC | ND | TT | TT | TC | TT | TT | TT |
| rs6820230 | G>A | p.Ala17 Thr | GG | AA | GA | GA | GG | GG | GG | GA | AA | AA | GG | GA | AA | GA | AA | GG | GA | GG | GG | GG | GG | GG | GA | nd | GG | GG | GA | GG | GG | GG |
| rs2276961 | G>A | p.Gly25 Arg | AA | GG | GA | GA | GA | GA | GG | GA | GG | GG | AA | GA | GG | GA | GG | nd | GA | AA | GA | AA | AA | nd | GA | GA | AA | AA | GA | AA | AA | nd |
| rs13113918 | T>C | p.Leu 79 Leu | CC | TT | TC | TC | TC | TC | CC | TT | TT | TT | CC | TC | TC | CC | TC | TC | TC | CC | TC | TC | CC | CC | CC | CC | CC | CC | CC | CC | CC | CC |
| rs10939650 | G>A | p.Thr 96 Thr | AA | GG | GA | GA | GA | GA | AA | GG | GG | GG | AA | GA | GA | AA | GA | GA | GA | AA | GA | GA | AA | AA | AA | AA | AA | AA | AA | AA | AA | AA |
| rs13125646 | T>C | p.Leu 160 Leu | TT | TC | TC | TT | TC | TC | TC | TT | TT | TC | TC | TC | TC | CC | TC | TC | TC | TC | TC | TC | CC | CC | CC | CC | CC | CC | CC | CC | CC | CC |
| rs16890979* | G>A | p.Val 253 Ile | AA | GA | AA | AA | GA | AA | GA | AA | AA | GG | GA | GA | GA | GA | GA | GA | GA | GA | GA | GA | GG | GG | GG | GG | GG | GG | GG | GG | GG | GG |
| rs3733591 | G>A | p.Arg 265 His | GG | GG | GG | GG | GG | GG | GG | GG | GG | GG | GG | GA | GG | GG | GG | GG | GA | GG | GG | GG | GA | GA | GG | GG | GG | GA | GG | GG | GG | GA |
| rs2280205 | C>T | p.Pro 321Leu | CT | CT | TT | TT | CC | CT | CC | CC | TT | CC | TT | CT | CC | CT | CT | CT | CT | CT | CC | CT | CT | CT | CT | CT | TT | CT | CC | TT | TT | CT |
