# Supplementary material for: The GLUT9 Gene Is Associated with Serum Uric Acid Levels in Sardinia and Chianti Cohorts
Source: PLoS Genet. 2007 Nov 9;3(11):e194. doi: 10.1371/journal.pgen.0030194 (PMC2065883; doi:10.1371/journal.pgen.0030194)
Supplement: Supplementary file 4 [file pgen.0030194.st004.doc]

**Supplementary Table 4**

**Numbers of individuals with each genotype for two SNPs associated with *GLUT9* in SardiNIA and InCHIANTI population cohorts**

rs6855911 SNP

|  | **Genotype Counts** | | |
| --- | --- | --- | --- |
|  | A/A | A/G | G/G |
|  |  |  |  |
| **SardiNIA** |  |  |  |
| All | 715 | 508 | 87 |
| Men | 322 | 246 | 47 |
| Women | 393 | 262 | 40 |
|  |  |  |  |
| **InCHIANTI** |  |  |  |
| All | 706 | 485 | 85 |
| Men | 327 | 205 | 40 |
| Women | 379 | 280 | 45 |

**rs7442295 SNP**

|  | **Genotype Counts** | | |
| --- | --- | --- | --- |
|  | A/A | A/G | G/G |
|  |  |  |  |
| **SardiNIA** |  |  |  |
| All | 882 | 455 | 55 |
| Men | 403 | 222 | 28 |
| Women | 479 | 233 | 27 |
|  |  |  |  |
| **InCHIANTI** |  |  |  |
| All | 764 | 443 | 68 |
| Men | 346 | 196 | 31 |
| Women | 418 | 247 | 37 |
